# Regulatory and Effector Cell Disequilibrium in Patients with Acute Cellular Rejection and Chronic Lung Allograft Dysfunction after Lung Transplantation: Comparison of Peripheral and Alveolar Distribution

**DOI:** 10.3390/cells10040780

**Published:** 2021-04-01

**Authors:** Laura Bergantini, Miriana d’Alessandro, Elda De Vita, Felice Perillo, Antonella Fossi, Luca Luzzi, Piero Paladini, Anna Perrone, Paola Rottoli, Piersante Sestini, Elena Bargagli, David Bennett

**Affiliations:** 1 Respiratory Disease and Lung Transplant Unit, Respiratory Diseases and Transplant Unit, Department of Medical Sciences, Surgery and Neurosciences, Siena University, 53100 Siena, Italy; dalessandro.miriana@gmail.com (M.d.); devita11@student.unisi.it (E.D.V.); felice.perillo87@gmail.com (F.P.); antonella.fossi@gmail.com (A.F.); a.perrone@ao-siena.toscana.it (A.P.); paola.rottoli@unisi.it (P.R.); sestini@unisi.it (P.S.); bargagli2@gmail.com (E.B.); david.btt@gmail.com (D.B.); 2 Thoracic Surgery Unit, Department of Medicine, Surgery and Neuroscences, Siena University Hospital, 53100 Siena, Italy; dr.luca.luzzi@gmail.com (L.L.); piero.paladini@unisi.it (P.P.)

**Keywords:** lung transplantation, acute rejection, chronic lung allograft dysfunction, bronchiolitis obliterans syndrome, flow cytometry, regulatory cells, effector T cells

## Abstract

Background: The immune mechanisms occurring during acute rejection (AR) and chronic lung allograft dysfunction are a challenge for research and the balance between effector and regulatory cells has not been defined completely. In this study, we aimed to elucidate the interaction of effector cells, mainly Th17, Th1 and Th2, and regulatory cells including (CD4^+^CD25^+^CD127^low/−^) T reg cells and phenotypes of B regs, CD19^+^CD24^hi^CD38^hi^, CD19^+^CD24^hi^CD27^hi^ and CD19^+^CD5^+^CD1d^+^. Methods: Bronchoalveolar lavage cells (BAL) and peripheral blood mononuclear cells (PBMCs) from stable lung transplanted (LTx )subjects (*n* = 4), AR patients (*n* = 6) and bronchiolitis obliterans syndrome (BOS) (*n* = 6) were collected at the same time. Cellular subsets were detected through flow cytometry. Results: A predominance of Th17 cells subtypes in the PBMCs and BAL and a depletion of Tregs, that resulted in decrease Treg/Th17 ratio, was observed in the AR group. CD19^+^CD24^hi^CD38^hi^ Bregs resulted increased in BAL of AR patients. Th1 cells predominance and a reduction of Tregs cells was observed in BAL from AR patients. Moreover, multivariate analysis showed interdependences within studied variables revealing that effector cells and regulatory cells can effectively discriminate patients’ immunological status. Conclusions: In AR, BOS and stable lung transplant, regulatory and effector cells clearly demonstrated different pathways of activation. Understanding of the balance of T cells and T and B regulatory cells can offers insights into rejection.

## 1. Introduction

Lung transplant (LTx) is the final therapeutic option for a variety of end-stage pulmonary diseases [1,2,3]. Advances in surgical techniques and immunosuppressive therapy have improved survival after transplant; however, acute rejection is still a common complication [4,5]. At mid-long term, chronic allograft dysfunction (CLAD) represents the leading cause of death; bronchiolitis obliterans syndrome (BOS) is the most common form of CLAD. Another restrictive form, termed restrictive allograft syndrome (RAS), was recently recognized [6].

BOS is characterized by chronic inflammation of the small airways and obliterative fibrosis, whereas peripheral lung tissue remains relatively unchanged [7]. The exact pathogenesis of these phenomena and the best clinical approach remain unclear [8]. The immune mechanisms occurring during lung graft dysfunction are a challenge for research. The implications of Th17 cells and related pro-inflammatory cytokines in AR and BOS were recently investigated and a close association between these cells and regulatory cells emerged [9]. Th17 cells and associated cytokines are recognized as key players in inflammation and autoimmune diseases [10,11]. Regulatory cells include regulatory T cells (Tregs) and regulatory B cells (Bregs), which play important roles in immune modulation [12,13,14]. Tregs seemed to downregulate Th17 effector function, while inhibition of Th17 function has been proposed as a target therapy in several diseases, including cancer [15].

New subsets of B cells with regulatory properties, described as CD19^+^CD1d^hi^CD5^hi^ [16], CD19^+^CD24^hi^CD38^hi^ [17] and CD19^+^CD24^hi^CD27^hi^ [18], were recently discovered. These cells are proposed to play a key role in homeostasis after lung transplant and can have a role in clinical setting as well: Breg features may help identify tolerant patients, making it possible to reduce immunosuppressants or withdraw drugs [19].

In this study, we quantified the percentage of Th17, Treg and Breg cell subsets in patients with AR and BOS and in Stable patients after LTx. We looked for relationships and mechanisms involving effector cells, mainly Th17 cells, and tolerance, quantified in terms of Bregs and Tregs, comparing the contemporary distribution of these cells in bronchoalveolar lavage (BAL) fluid and peripheral blood.

## 2. Materials and Methods

### 2.1. Study Design and Population

Sixteen consecutive LTX patients who underwent bronchoscopy with BAL and transbronchial biopsy for clinical purpose, and whose peripheral blood mononuclear cells were collected, were included in the study. The cohort was divided into three groups according to diagnosis at time of sample collection: 6 patients with BOS (4 male, age 52.3 ± 14.4), 6 patients with AR (4 male, age 50.6 ± 14) and 4 patients who were considered stable (1 male, age 50.5 ± 8). The demographic characteristics of the patients are reported in Table 1. 

The three groups were matched for age and sex. Flow cytometric analysis of cells was performed for comparison of the three groups. Diagnosis of AR and BOS was performed according to established guidelines [20,21]. Antibody-mediated rejection (AMR) was excluded for all patients. All patients received corticosteroid therapy at surgery consisting of intravenous methylprednisolone 125 mg before graft reperfusion followed by 375 mg on day 0 and tapering from 1 mg/kg on day 1. Induction therapy was administered to all patients with basiliximab (20 mg on day 0 and day 4). Tacrolimus was introduced on days 3–5. Azathioprine 100 mg/day or mycophenolate mofetil 1 g/day was introduced in most patients from day 7 to day 10.

### 2.2. Lung Function Tests

The following lung function parameters were recorded according to ATS/ERS standards using a Jaeger body plethysmograph with corrections for temperature and barometric pressure: forced vital capacity (FVC), forced expiratory volume in the first second (FEV1), diffusing capacity of the lung for carbon monoxide (DLCO) and FEV1/FVC ratio. All were expressed as percentages of predicted values.

### 2.3. Preparation and Storage of PBMCs

Preparation and storage of peripheral blood mononuclear cells (PBMCs) was performed at the laboratory of the Respiratory Diseases Unit, University Hospital of Siena, Italy, from January 2020 to May 2020. The peripheral blood samples were collected after 8-h fasting in a tube containing EDTA antiipfulant (BD Vacutainer^®^ EDTA tubes, BD Biosciences, CA, USA) and processed within 8 h. Briefly, a layer of blood was added to 15 mL Ficoll Histopaque^®^-1077 (Sigma-Aldrich, INC., It, UE, Saint Louis, MO, USA) in a conical 50 mL tube and centrifuged for 30 min at 3000 rpm in a swinging-bucket rotor without brake. The mononuclear cell layer was transferred to a new conical 50 mL tube (Corning^®^ 50 mL centrifuge tubes, Sigma-Aldrich, INC., It, UE, Saint Louis, MO, USA), adding 15 mL RPMI 1640 medium (Gibco^®^, Thermo Fisher Scientific, Inc., It, UE, Waltham, MA, USA), and centrifuged at 1500 rpm for 10 min. Supernatant was carefully removed and the cells were stored in liquid nitrogen until analysis.

## 3. Bronchoalveolar Lavage Cell Processing and Collection

Bronchoscopy with BAL was performed for diagnostic reasons. BAL fluid was obtained by instillation of four 60-mL aliquots of saline solution by fibrobronchoscope (Olympus IT-10, Olympus Medical System, It, Haryana, India) wedged in a subsegmental bronchus of the middle lobe or lingula. The first sample was kept separate from the others and was not used for immunological tests. Briefly, BAL was filtered through sterile gauze and cell count was determined by cytocentrifuge smear (600 rpm for 5 min) with a Diff quik stain kit (DiaPath, Italy); a total of 500 cells were counted. Cell viability was determined by Trypan blue exclusion in a Burker Chamber. Resting samples were centrifuged (1500 rpm for 10 min). Supernatant was carefully removed and the cells stored in liquid nitrogen until analysis.

### 3.1. Lymphocyte Immunophenotyping by Flow Cytometry

The samples were processed by flow cytometry using a panel of monoclonal antibodies (BD Multitest™ 6-color TBNK, San Jose, CA, USA), including fluorescein isothiocyanate-labeled (FITC) CD3, phycoerythrin-(PE) labeled CD16 and CD56, PerCP-Cy5.5-labeled CD45, PE-Cy7-labeled CD4, allophycocyanin (APC)-labeled CD19 and (APC)-Cy7-labeled CD8 according to the manufacturer’s instructions. At least 50,000 events were collected for each sample. Data were analyzed using Kaluza Analysis 2.1 (Beckman and Coulter life sciences, Indianapolis, IN, USA). Lymphocytes were distinguished on the basis of forward (FSC) versus side (SSC) scatters and additional gating was applied using SSC versus CD45 to distinguish lymphocytes from cell debris. Specific panels were subsequently assessed to identify T lymphocytes, B lymphocytes and NK cells. T lymphocyte subpopulations were gated in order to distinguish CD3^+^CD4^+^ (T-helper), CD3^+^CD8^+^ (T-cytotoxic) and CD3^+^CD16/56^+^ (NKT-like) cells.

### 3.2. Th17 Phenotyping by Flow Cytometry

Multicolor flow cytometric analysis was performed using the following fluorochrome-labeled monoclonal anti-human antibodies: CD4-APC-Vio770 (REA623), CD45RA-PE-Vio770 (REA562), CD196 (CCR6)-APC (REA190), CD183 (CXCR3)-VioBright FITC (REA232) and CD194 (CCR4)-PE (REA279) (all from Miltenyi Biotec, Bergisch Gladbach, Germany).

Cells were stained for 30 min at 4 °C, measured with a Facs CantoII flow cytometer and analyzed using Kaluza Analysis 2.1 (Beckman and Coulter life sciences Indianapolis, Indiana, USA). Lymphocytes were distinguished on the basis of forward (FSC) versus side (SSC) scatters and additional gating was applied using SSC versus CD4 to distinguish lymphocytes from cell debris.

Th1, Th17 and Th1-like Th17 cells were defined based on the markers CD45 RA and CCR6, with and without expression of CXCR3 and CCR4. 

For analysis, total CD4^+^CD45^−^ cells were divided according to expression of CCR6. CCR6^+^ cells were classified as CXCR3^+^CCR4^+^ (Th17 double-positive), CXCR3^−^CCR4^+^ (Th17) and CXCR3^+^CCR4^−^ (Th1-like Th17), whereas CCR6^−^ cells were classified as CXCR3^−^CCR4^+^ (Th2) and CXCR3^+^CCR4^−^ (Th1) [22].

## 4. Regulatory T Cell Detection by Flow Cytometry

Multicolor immunofluorescent staining, followed by flow cytometric analysis, was used to determine the phenotype of regulatory T and B cells. Blood samples were processed by flow cytometry using a panel of monoclonal antibodies (BD Human Regulatory T Cell Cocktail, San Jose, CA, USA), including FITC anti-Human CD4 (clone SK3), PE-Cy7 anti-Human CD25 (clone 2A3) and Alexa Fluor^®^ 647 anti-Human CD127 (clone HIL-7R-M21). Lymphocytes were distinguished on the basis of forward (FSC) versus side (SSC) scatters and additional gating was applied using SSC versus CD4. A secondary dot-plot was subsequently assessed to identify CD25^bright^CD127^−/low^ Tregs.

## 5. Regulatory B Cell Detection by Flow Cytometry

The following anti-human mAbs were used to detect regulatory B cells (Bregs) as surface markers: FITC-conjugated anti-CD38, PE-conjugated anti-CD1d, PE-Cy7-conjugated anti-CD19, PerCP-CY5.5-conjugated CD5, APC-H7-conjugated anti-CD24 and BV510-conjugated anti-CD27. Lymphocytes were distinguished on the basis of forward (FSC) versus side (SSC) scatters and additional gating was applied using SSC versus CD19 to distinguish lymphocytes from cell debris. Three different subtypes of CD19+ Bregs were divided on the basis of CD24^hi^CD38^hi^, CD24^hi^CD27^hi^ and CD5^+^CD1d^+^. At least 1,000,000 events were read by flow cytometer for each sample. The data were analyzed using DIVA software (BD Biosciences, San Diego, CA, USA).

### Statistical Analysis

The results were expressed as means and standard deviations (SD) or medians and quartiles (25th and 75th percentiles) for continuous variables as appropriate. One-way ANOVA non parametric test (Kruskal-Wallis test) and Dunn test were performed for multiple comparisons. The Chi-squared test was used for categorical variables. The Spearman test was used to look for correlations. A *p* value less than 0.05 was considered statistically significant. Statistical analysis was performed by SPSS Software (SPSS Inc., Chicago, IL, USA) and graphic representation of the data by GraphPad Prism 8.0 software (Graphpad Holdings, LLC). Unsupervised Principal component analysis (PCA) was employed to reduce the dimensionality of data hyperspace and Hierarchical Heatmaps for sample clustering based on their cellular composition. The data matrix with variance was constructed with Microsoft Excel and PCA with heatmap was performed using BioVinci software (BioTuring Inc., San Diego, CA, USA) and ClustVis (http://biit.cs.ut.ee/clustvis/, accessed on 28 March 2021) Software. The cellular subsets of patients were also employed to create a decision tree model for the detection of best clustering variables through the Gini criterion.

## 6. Results

### 6.1. Population

Demographics and clinical features of our 16 patients are shown in Table 1, including sex, age at transplant, indications for LTx, type of transplant, length of follow-up and type of immunosuppressant therapy used. Lung function tests showed FEV1s (% of predicted) (median (*IQR*)) of 78 (60–81), 64 (57–94) and 53 (40–63), in Stable, AR and BOS groups, respectively. The same trends were observed for FVC (% of predicted) (median (*IQR*)): 75.5 (73–78), 72 (58–94) and 67 (59–79), respectively, and for DLCO (% of predicted) (median (*IQR*)): 55 (51–60), 41 (30–58) and 55 (44–52), respectively, albeit without significant differences between groups.

### 6.2. Peripheral Blood Mononuclear Cell Analysis

**Figure 1:**
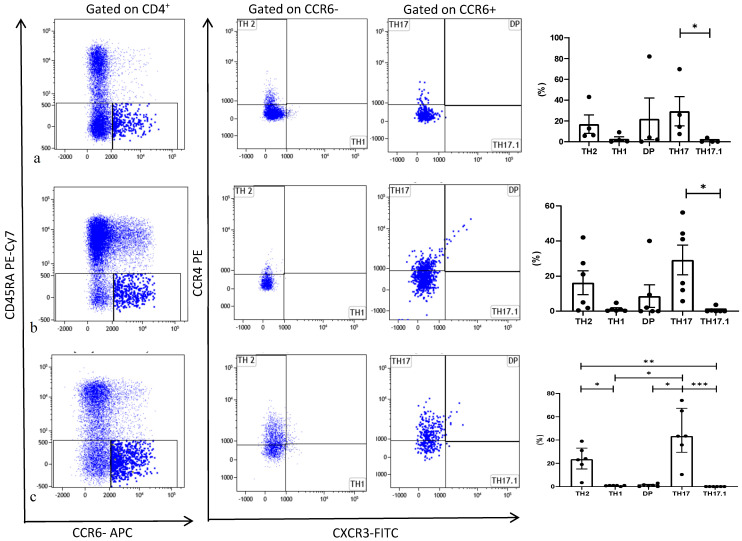
Representative dot plots related to the expression of different chemokine receptors gated in gated CD4^+^. T from Stable (**a**), BOS (**b**) and AR (**c**). Three experiments (one for Stable, one for BOS and one for AR) out of 16 are shown. Data represent individual values (dots), mean (center bar) ± SEM (upper and lower bars). If not indicated, *p* value is not significant. * *p* < 0.05, ** *p* < 0.01, *** *p* < 0.001.

The different PBMC subsets are shown by group in Table 2. Th cell distribution in the three groups is reported in Figure 1. Among stable patients, Th cells showed a slight increase in percentages of Th17 cells with respect to Th17.1 (Figure 1a). In the BOS group, Th cells showed increased Th17 cell percentages with respect to Th1 and double-positive cells (Figure 1b). In the AR group, the Th17 (CCR4^+^CXCR3^−^) subtype showed increased percentages with respect to the other Th cells (including Th1, Th2, Th17.1 and double-positive). Th2 cells also showed a statistically significant increase with respect to Th1 and Th17.1 (Figure 1c) among patients in the AR group. The percentages of CD4^+^, CCR6^−^ and CCR6^+^ did not differ significantly between groups.

**Figure 2:**
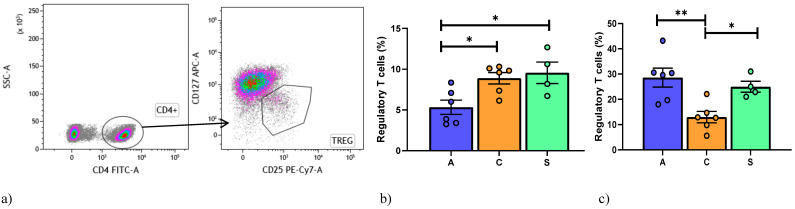
(**a**) Gating strategy used to analyze markers for Regulatory T cells detection. Treg are CD127^−^CD25. (**b**) Data represent individual values on PBMC, mean (center bar) ± SEM (upper and lower bars). If not indicated, *p* value is not significant. * *p* < 0.05, ** *p* < 0.01. (**c**) Data represent individual values on BAL samples, mean (center bar) ± SEM (upper and lower bars). Statistical analysis by Mann–Whitney nonparametric test; if not indicated, *p* value is not significant. * *p* < 0.05, ** *p* < 0.01. Abbreviations: A: acute, C: chronic, S: stable.

Regarding regulatory T cells, a gate strategy was reported in Figure 2a. Lower percentages of CD4^+^CD25^+^CD127^−/low^ Treg were observed in the AR than in the BOS and Stable groups (Figure 2b).

**Figure 3:**
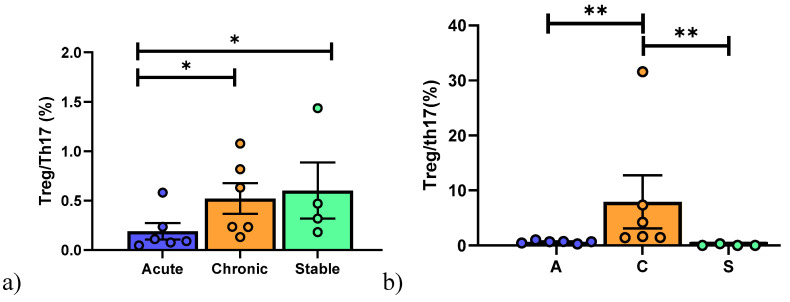
(**a**) Data represent individual values on PBMC, mean (center bar) ± SEM (upper and lower bars). If not indicated, *p* value is not significant. * *p* < 0.05, ** *p* < 0.01. (**b**) Data represent individual values on BAL samples, mean (center bar) ± SEM (upper and lower bars). If not indicated, *p* value is not significant. * *p* < 0.05, ** *p* < 0.01. Abbreviation: A: acute, C: chronic, S: stable.

Th1/Th2, Treg/Th17 and Th1/Treg data are reported in Table 2. In the BOS group, the Th1/Th2 ratio was higher than in Stable and AR patients (*p* = 0.03; *p* = 0.04, respectively) and the Treg/Th1 ratio lower in AR group than in BOS and Stable patients (*p* = 0.005; *p* = 0.002, respectively). As expected, the Treg/Th17 ratio of the AR group was significantly different to that of the other groups (Figure 3a).

**Figure 4:**
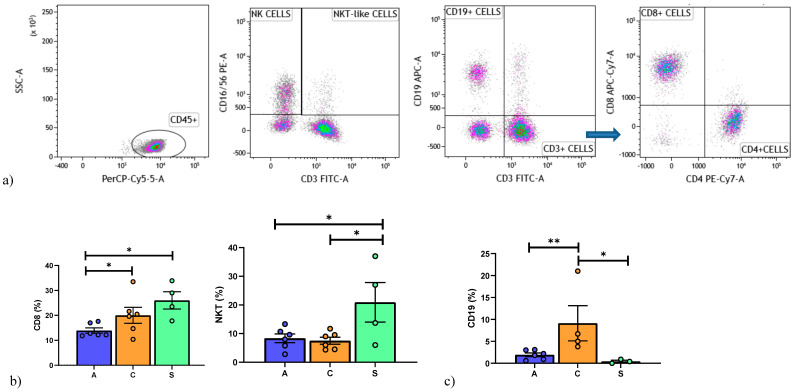
(**a**) Gating strategy used to analyze T cell subsets. (**b**) Data represent individual values on PBMC samples, mean (center bar) ± SEM (upper and lower bars). If not indicated, *p* value is not significant. * *p* < 0.05, ** *p* < 0.01. (**c**) Data represent individual values on BAL samples, mean (center bar) ± SEM (upper and lower bars). * *p* < 0.05, ** *p* < 0.01. Abbreviations: A: acute, C: chronic, S: stable.

AR patients showed fewer CD8^+^ T cells than BOS and Stable patients, whereas more NKT-like cells were detected in the Stable group than in the other two groups (Figure 4b).

**Figure 5:**
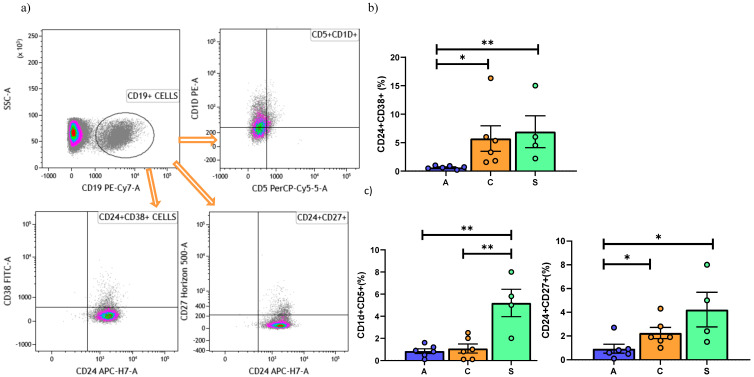
(**a**) Gating strategy used to analyze B reg cells detection. (**b**) Data represent individual values on PBMC, mean (center bar) ± SEM (upper and lower bars). If not indicated, *p* value is not significant. * *p* < 0.05, ** *p* < 0.01. (**c**) Data represent individual values on BAL, mean (center bar) ± SEM (upper and lower bars). If not indicated, *p* value is not significant. * *p* < 0.05, ** *p* < 0.01. Abbreviations: A: acute, C: chronic, S: stable.

The analysis of the three main phenotypes of Bregs found that CD24^hi^CD38^hi^ Bregs were lower in the AR than in the BOS and Stable groups (Figure 5b). No significant differences in the expression of CD24^hi^CD27^hi^ and CD5^+^CD1d^+^ were observed among the three groups.

### 6.3. Broncho-Alveolar Lavage Cell Analysis

**Figure 6:**
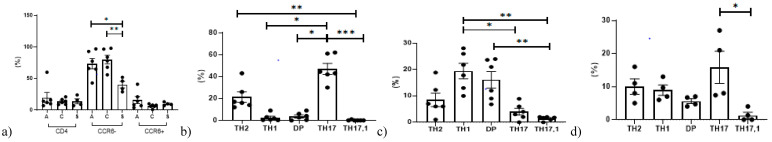
(**a**) CD4^+^, CCR6^−^ and CCR6^+^ cells differed in percentages between the Stable group and the other two groups. Data represent individual values on BAL, mean (center bar) ± SEM (upper and lower bars). If not indicated, *p* value is not significant. * *p* < 0.05, ** *p* < 0.01, *** *p* < 0.001. Th cells distribution on BAL of Acute Rejection group (**b**), BOS (**c**) and Stable patients (**d**).

Table 3 shows our comparison of the different BAL cell subsets in our patients. CD4^+^CCR6^−^ cells differed in percentages between the Stable group and the other two groups (Figure 6a). In the AR group, analysis of Th cells revealed a predominance of Th17 with respect to Th1, Th17.1 and double-positive cells. Th2 were also significantly more abundant than Th17.1 cells (Figure 6b).

In the BOS group, analysis of Th cells showed higher percentages of Th1 cells than Th17 and Th17.1 cells. In the BOS group a difference was also observed between the abundance of double-positive and Th17.1 cells (Figure 6c).

Among Stable patients, the analysis of Th cells showed a homogeneous distribution of the subsets, differing only for Th17 and Th17.1 cells (Figure 6d).

**Figure 7:**
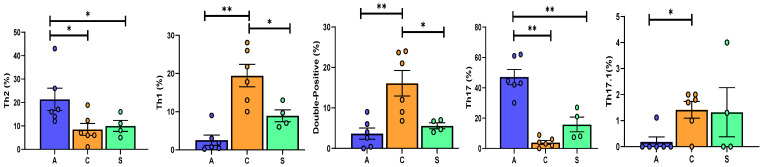
Data represent individual values on BAL, mean (center bar) ± SEM (upper and lower bars). If not indicated, *p* value is not significant. * *p* < 0.05, ** *p* < 0.01. Abbreviations: A: acute, C: chronic, S: stable.

Th2 and Th17 cells were significantly higher in the AR than in the other two groups, whereas we found a predominance of Th1 and double positive cells in the BOS group. Interestingly, Th17.1 cells differed in percentage between the BOS and AR groups (Figure 7).

Regarding Tregs, the BOS group showed lower percentages of CD4^+^CD25^+^CD127^−/low^ than the Stable and AR groups (Figure 2c).

Th1/Th2, Th17/Treg and Th1/Treg ratios are reported in Table 3. In BOS patients, the Th1/Th2 ratio was higher than in the other groups (*p* = 0.002; *p* = 0.004, respectively). On the contrary, Treg/Th1 was significantly higher in the AR than the BOS and Stable groups (*p* = 0.0001; *p* = 0.006, respectively), whereas Treg/Th17 was higher in BOS patients (Figure 3b).

B cells (CD19^+^) were higher in BOS patients than the other groups (Figure 4c). Regarding Bregs, interestingly, lower percentages of CD5^+^CD1d^+^ in the AR and BOS group than in Stable patients was observed, while CD24^+^CD27^+^ showed decrease percentages in the AR than in Stable and BOS patients (Figure 5c).

### 6.4. BAL and Peripheral Blood Comparison of Subpopulations

**Figure 8:**
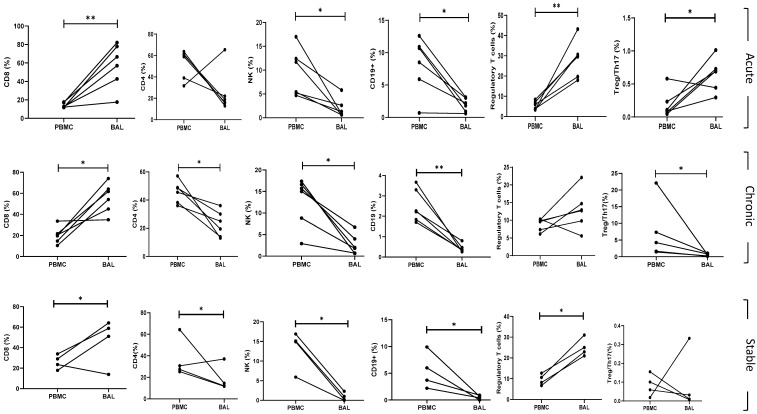
Data represent comparison analysis on BAL and PBMC, mean (center bar) ± SEM (upper and lower bars). If not indicated, *p* value is not significant. * *p* < 0.05, ** *p* < 0.01.

When we compared BAL and peripheral blood findings, higher percentages of CD8 cells and Tregs were found in BAL, whereas CD4, NK and CD19 were lower in BAL than in blood in all three groups (Figure 8). Th cells did not differ between BAL and blood in the Stable group. Regarding Bregs, CD24^hi^CD38^hi^ were lower in blood of the AR group than in BAL (*p* = 0.006).

#### Multivariate Analysis

**Figure 9:**
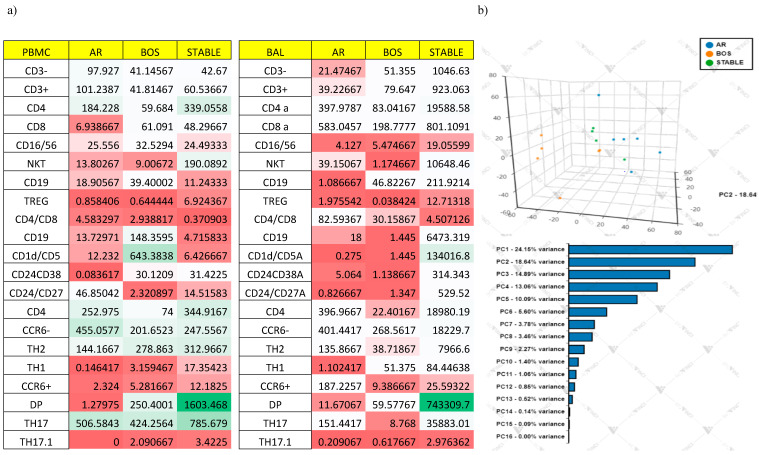

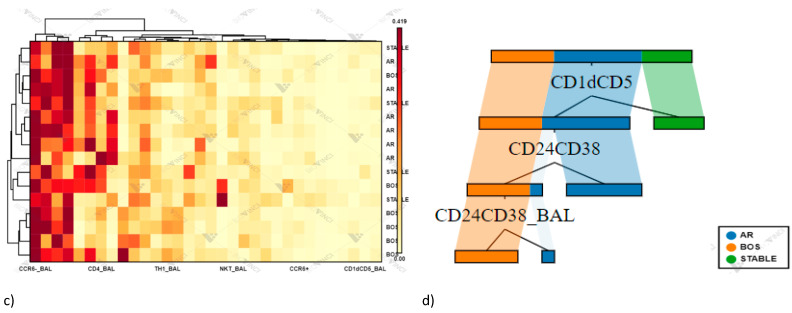
(**a**) Interindividual variance (CV) for cell populations was calculated for each three conditions on PBMC and BAL samples. (**b**) Principal Component Analysis (PCA) of cells. (**c**) The heat map analysis. (**d**) The cellular subsets of patients were employed to create a decision tree model for the detection of best clustering variables.

Variance analysis revealed that BAL cellular distribution appeared homogeneous within condition, yielding lower interindividual variance (red boxes) for many of the cellular populations analyzed, while more significant differences when comparing PBMC cellular distribution, yielding higher interindividual variance (green boxes) for many of the cellular populations analyzed (Figure 9a).

PCA plots showing effect for each sample on cellular variation in these three conditions: AR, (*n* = 6) BOS (*n* = 6) and Stable (*n* = 4). The first PC and second PC resolved 24.15% and 18.64% of the total variance, respectively (Figure 9b). The heat map is based on hierarchical clustering applied for row and columns and reported as Euclidean distance. The color of the heat varies from white indicating relative under-representation to red indicating relative over-representation. Clusters are sorted according to adjusted *p* values, so that the cluster at the top shows the most significant abundance changes between the three conditions (Figure 9c).

The cellular subsets of patients were employed to create a decision tree model for the detection of best clustering variables by Gini criterion. The best population is CD1Dcd5Breg followed by CD24^+^CD38^+^Breg on PBMC and CD24^+^CD38^+^Breg on BAL (Figure 9d).

Supplementary Figure S1 shows the different spatial positions of the PCA 2D plot for lymphocytes subsets detected through flow cytometry in BAL samples. The eigenvalue percentage significance of the first three PCs was 59.4%. Supplementary Figure S2 shows the different spatial positions of the PCA 2D plot for lymphocytes subsets detected through flow cytometry in PBMC samples. The eigenvalue percentage significance of the first three PCs was 60.8%. Intraclass dispersion occurred mainly along the first and second PCs for all analysis.

Concerning analysis of BAL cells, high homogeneity was observed in the AR group and less homogeneity in the Stable group. Intraclass dispersion occurred mainly along the first and second PCs. For PBMCs, less homogeneity was observed with respect BAL samples. Intraclass dispersion occurred mainly along the first and third PCs.

### 6.5. Correlation of Lung Function Parameters and Immune Cells 

Spearman correlation was performed between lung function parameters and between peripheral blood and BAL cell subtypes. Interestingly, in PBMC of the AR group, FVC percentages showed direct correlations with NKT (r = 0.88, *p* = 0.03) and CCR6^+^ cells (r = 0.88, *p* = 0.03). In the BOS group, direct correlations emerged between CCR6^+^ and FVC (r = 0.94, *p* = 0.010) and between CCR6^+^ and DLCO (r = 0.99, *p* = 0.017). An inverse correlation between CD4 and FVC also emerged for BOS patients (r = 0.25 *p* = 0.03). 

## 7. Discussion

The role of different immunological networks in lung transplant patients has been investigated by many authors, including Th1, Th2, Th17, Tregs and some Bregs, but the complex immunological interactions between multiple cell phenotypes is not yet well understood. The aim of the present study was to obtain insights into the relationships and mechanisms linking effector cells and tolerance systems, systemically and locally in the lung allograft, by comparing expression of Th17, Treg and Breg cell subsets in peripheral blood and BAL of lung transplant patients who did and did not develop acute rejection or BOS.

In recent years, the importance of Bregs in the pathobiology of different diseases, including graft rejection and tolerance mechanisms has become evident [18]. Our study is the first to explore all Breg phenotypes and to report a decrease in the proportion of CD19^+^CD1d^hi^CD5^+^ and CD24^hi^CD27^hi^ Bregs in peripheral blood and BAL of patients with acute rejection, and an inverse pattern for CD19^+^CD24^hi^CD38^hi^ cells, which were higher in BAL cells and lower in PBMC.

CD19^+^CD1d^hi^CD5^+^ B cells inhibit Th17 responses [16,23], although no data have been available on their function in lung transplant recipients. Qin, J. et al. [16] found that the proportion of the CD19^+^CD1d^+^CD5^+^ Breg subset was significantly depressed in Graves’ disease. Zhang et al. [23] reported that these Breg cells were elevated in peripheral blood of patients with tuberculosis and inhibited Th17 cell-related cytokine function in these patients. Our findings in peripheral blood and BAL suggest similar mechanisms during acute rejection episodes, in which the decrease in CD19^+^CD1d^hi^CD5^+^ Bregs may participate in upregulation of the Th17 population.

Broos, C.E. et al. [22] investigated the role of CD19^+^CD24^hi^CD38^hi^ Bregs in lung transplant patients, suggesting association of this Breg phenotype with BOS, mycophenolate immunosuppression and concomitant infection. Interestingly, Flores-Borja, F. et al. [24] proposed a dual capacity of CD19^+^CD24^hi^CD38^hi^ Bregs in maintaining the pool of Tregs and Th1/Th17 populations through controlling over-production of proinflammatory cytokines, preventing engagement of CD4 with Th1 and Th17 and participating in converting effector T cells into Tregs. Our findings are in line with this data: CD19^+^CD24^hi^CD38^hi^ Bregs were downregulated in PBMC and were associated with an increase in Th17 and a decrease in Tregs. The inverse behavior of this subphenotype that we observed in BAL may be due to recruitment of these cells into the lung during acute rejection in an attempt to restore cell homeostasis. This intriguing hypothesis requires further study in a larger statistical sample.

CD19^+^CD24^hi^CD27^+^ Bregs contribute to immune response control principally by secreting IL-10 [25,26]. Interestingly, compared to CD19^+^CD24^hi^CD38^hi^, these cells showed distinct effects on T cells: Bregs with the immature cell marker CD38 induce development of Tregs by limiting differentiation of Th1 and Th17 cells [24], while Bregs with memory B cell marker CD27 are mainly responsible for suppressing activity of CD4^+^ T cells [27]. In our study, we observed a lower percentage of CD19^+^CD24^hi^CD27^+^ Bregs in BAL and peripheral blood in cases of acute and chronic rejection, sustaining evidence that these patients show a higher percentage of CD4^+^ cells.

Our results are in line with literature that also focused on the crosstalk between effector functions and tolerance systems [24]. Patients in the acute rejection group showed a predominance of Th17 cells, both in BAL and peripheral blood, and simultaneous impairment of Tregs resulting in a depressed Treg/Th17 ratio. Tregs, Th17 cells and their balance are considered central for the homeostasis of immune responses and tolerance [28,29]. An imbalance of these cells is closely linked to the pathogenesis of various diseases and conditions, including autoimmunity, transplant rejection and carcinogenesis [30,31]. The role of Tregs and Th17 cells in lung rejection has been widely investigated [32] and the networks involving them have been proposed in a new immunological approach to transplantation [33]. Neujahr, D.C. et al. [5] reported low Treg frequencies in BAL in the aftermath of acute rejection. Recently, Ius, S. et al. [34] demonstrated that a higher frequency of Tregs early after lung transplant is protective against CLAD and associated with better survival. Piloni, D. et al. [35] reported that variations in peripheral CD4^+^CD25^hi^CD127^−/low^ Treg counts are predictive of CLAD onset/progression.

Concerning Th17 analysis, our results showed significantly higher percentages of Th17 cells in acute rejection patients than in the other two groups, both in BAL and peripheral blood.

BAL isconsidered a biological fluid representative of the alveolar airway compartment that provides important information on the complex immunopathogenesis of lung disorders [36,37,38].

In our study, BAL Tregs reflected the immunological environment occurring after lung transplant better than PBMC. In fact, a significantly lower percentage of BAL Tregs was observed in BOS and acute rejection patients than in Stable patients. On the contrary, in peripheral blood, clear depletion of these cells only emerged in cases of acute rejection.

We also explored the potential role of the Th17.1 cell line after lung transplant, observing significantly higher percentages of these cells in BAL of patients with BOS than in the other groups. These cells can release IFN-γ, and although phenotypically characterized by surface expression of CCR6, a marker of Th17 cells [39], they have been demonstrated to play a key role in the development of autoimmune diseases and pulmonary sarcoidosis, where their enhancement in BAL has been correlated with the onset of chronic disease [40,41]. No data on solid organ transplant are currently available, making our study the first to attempt to evaluate the role of Th17.1 cells in lung transplant patients.

Our results demonstrated the activation of a complex cellular and cytokine network in the different conditions examined and allow us to distinguish specific patterns among regulatory and effector cells that could represent potential therapeutic targets that need further investigation.

PCA analysis clearly showed interdependences within studied variables, revealing that these cell phenotypes were able to discriminate the immunological status of lung transplant patients; in particular, cellular analysis of BAL cells greatly differentiated acute cellular rejection and chronic lung allograft dysfunction from Stable patients. Decision tree analysis also reported the discriminatory variables; in particular peripheral CD19^+^CD1d^hi^CD5^+^, Bregs seemed to clearly discriminate Stable patients from BOS and AR.

Instead, peripheral CD19^+^CD24^hi^CD38^hi^ further discriminate BOS from AR. The limits of the study include its monocentric and retrospective design and the small sample size and lack of prior research on the topic. Moreover, the analysis is almost exclusively based on the expression of antigens and not on the actual functional activity of a given cellular subsets. The relative released cytokines for each cellular subset are not evaluated as well as intracytoplasmic identification of this cytokine. However, our patients were carefully characterized and our complex experimental model, including BAL and blood cells, firstly evaluated contemporarily the peripheral and lung graft environments and then analyzed the composite immune and cell networks of lung transplant patients. A deeper analysis of the cell populations identified can be conducted.

## 8. Conclusions

In acute and chronic graft rejection and stable lung transplant patients, regulatory and effector cells clearly demonstrated different pathways of activation. Understanding the complex crosstalk between T cells and B cells, both in at alveolar and peripheral level, is necessary to improve the biology of these cells during rejection after LTX. The balance of T cells and regulatory cells, including Tregs and new phenotypes such as Bregs, can offer insights into allograft rejection. In this study, a discriminatory effect of Breg emerged for distinguish patients with acute and chronic rejection from stable LTX patients. Further studies investigating new therapeutic approaches aimed to expand these cellular compartments for the induction of graft tolerance are needed.

## Figures and Tables

**Table 1:** Demographic data of 16 enrolled patients. Abbreviations: M = male, F = female, CF: cystic fibrosis, IPF: idiopathic pulmonary fibrosis, HP: hypersensitivity pneumonitis, CPFE = combined pulmonary fibrosis and emphysema, COPD: chronic obstructive pulmonary disease, IPAF = Interstitial pneumoniae with autoimmune features, Sarc = sarcoidosis, PLCH = pulmonary langherans cells histiocytosis. B = Bilateral, S = single, Pred = Prednisone, Tac = tacrolimus, MMF = mycophenolate mofetil, AZA = Azathioprine.

Number of Patients/Sex	Age (yr)	Tx Indication	Type of TX	Months from Tx	Immunosuppression at Enrollment	Clinical Conditions
1/M	39	CF	B	17	Pred + Tac + MMF	AR
2/F	63	IPF	S	7	Pred + Tac + MMF	AR
3/M	62	IPF	S	2	Pred + Tac + MMF	AR
4/F	38	CF	B	10	Pred + Tac	AR
5/M	37	CF	B	2	Pred + Tac	AR
6/M	65	HP	S	3	Pred + Tac + MMF	AR
7/M	60	CPFE	B	29	Pred + Tac	BOS
8/M	64	COPD	S	9	Pred + Tac + MMF	BOS
9/F	33	CF	B	9	Pred + Tac + MMF	BOS
10/M	68	IPAF	S	22	Pred + Tac + MMF	BOS
11/F	37	CF	B	84	Pred + Tac + MMF	BOS
12/M	52	HP	S	43	Pred + Tac + MMF	BOS
13/F	44	SARC	B	5	Pred + Tac	S
14/F	46	CF	B	84	Pred + Tac + AZA	S
15/F	50	PLCH	B	7	Pred + Tac + MMF	S
16/M	62	IPF	B	9	Pred + Tac	S

**Table 2:** Cellular analysis on peripheral blood mononuclear cells (PBMCs) in AR, BOS and Stable group.

PBMCs	AR	BOS	STABLE	*p* Value
Lymphocytes phenotyping:				
CD3^+^:	76.8 (72–89)	75.6 (72.6–79.9)	77.9 (74.9–79.6)	ns
− CD4 − CD8 − NKT − CD4/CD8	59.6 (44–63.7)	47 (40–48.8)	28.9 (26.8–39)	ns
	12.5 (12.2–17.6)	20 (15.9–21.3)	26.2 (22–30.2)	0.01
	8.8 (6.3–13.3)	7.5 (4.8–9.4)	20 (11.7–29.5)	ns
	3.6 (3.2–5.1)	2.2 (1.9–3)	1.4 (1.2–1.7)	<0.0001
CD3^−^:	17.9 (12.5–35.4)	22.2 (18.45–25.1)	19.4 (17.9–22.1)	ns
− CD19 − CD16/56	9.6 (6.5–12.6)	6.3 (4.2–11.3)	4.8 (3.3–6.9)	ns
	8.35 (5.25–17)	15.3 (10.3–16.3)	15 (12.6–15.6)	ns
Th cells subtypes:				
CD4^+^CCR6^−^:	41.1 (25.8–67.3)	46.2 (37.6–55.6)	51.5 (42–57.8)	ns
− Th1 − Th2 − Th1/Th2	1 (0.7–1)	0.7 (0.2–1)	0.9 (0.2–3.3)	ns
	23.5 (20–39)	13 (2.6–25)	9.5 (6.1–20)	ns
	0.03 (0.02–0.05)	0.11 (0.06–0.17)	0.04 (0.02–0.39)	ns
CD4^+^CCR6^+^:	4 (2.9–6.3)	2.15 (1.25–4.9)	3.25 (1.4–5.6)	ns
− Th17 − Th17.1 − DP	43.3 (37.6–74)	28.5 (13–43.4)	20 (12.5–36.8)	ns
	0 (0–0)	0 (0–0.3)	0 (0–0.9)	ns
	0.9 (1.8–3)	1 (0–7.53)	2.9 (1.4–23.5)	ns
Regulatory B subtypes:				
− CD24CD38 − CD24CD27 − CD1dCD5	0.65 (0.5–1)	4.3 (2.1–5.8)	5.2 (3.9–8.2)	0.0005
	1.16 (0.6–17)	1.8 (1.7–2.5)	7.4 (5.2–9.7)	ns
	2.45 (0.67–9.2)	2.5 (7.6–3.1)	2(1.9–3)	ns
Regulatory T cells:				
− Treg − Treg/Th17 − Treg/Th1	4.9 (3.5–8.3)	9.4 (7.8–11.1)	9.71 (7.9–10)	0.0003
	0.10(0.06–0.24)	0.1(0.08–0.14)	0.4 (0.28–0.71)	ns
	4.8(3.7–5.9)	16.4 (9.6–28.7)	14.4 (6.5–20.9)	0.004

**Table 3:** Cellular analysis on BAL in AR, BOS and Stable group.

BAL	AR	BOS	STABLE	*p* Value
Lymphocyte phenotyping:				
CD3^+^:	85.9 (82.9–88.9)	73.4 (69–79)	88 (75.3–87.2)	ns
− CD4 − CD8 − NKT − CD4/CD8	17.5 (15.7–21.2)	22.2 (15.4–28.7)	13.2 (11.9–20)	ns
	61.8 (46.2–75)	57.8 (47.2–63.4)	54.9 (41.7–60.1)	ns
	9.5 (3.3–14)	2 (1.1–2)	5.5 (4.3–7.1)	0.04
	3.0 (2.1–3.1)	1.28 (1–1.42)	0.22 (0.2–0.8)	0.0001
CD3^−^:	11.3 (7.4–13.6)	21.5 (15.2–25)	14.5 (11.4–21)	ns
− CD19 − CD16/56	2 (1.2–2.8)	4.5 (3.8–6.2)	0.5 (0.3–0.75)	0.0023
	1 (0.6–2.2)	1.9 (0.97–3.5)	0.6 (0.15–1.3)	ns
Th cells subtypes:				
CD4^+^CCR6^−^	71.7 (65.2–89.2)	81(70.5–93.5)	40 (32.7–47.5)	0.013
− Th1 − Th2 − Th1/Th2	0.8 (0.4–1.5)	19.8 (14.2–24.9)	8.35(6.7–10.5)	<0.0001
	16.5 (14.5–23.75)	6.5(6–10.9)	9.5 (7.2–12.2)	0.04
	0.04 (0.01–0.11)	2.26 (1.3–9.2)	0.8 (0.6–1.6)	<0.0001
CD4^+^CCR6^+^	11.3 (7.4–19.25)	6.9 (6–7.9)	9.4 (8.5–10.7)	ns
− Th17 − Th17.1 − DP	43.7 (42.2–56.8)	3.5 (2.2–5.5)	14.5 (7.8–22.5)	<0.0001
	0 (0–0)	1.75 (1.2–1.9)	0.65 (0–1.9)	ns
	1.6 (0.45–3.65)	16.5 (9.7–23)	5.7(4.5–6.7)	0.0014
Regulatory B subtypes:				
− CD24CD38 − CD24CD27 − CD1dCD5	3.3 (2.2–5.1)	1.85 (1.7–2.6)	2 (1.2–4.9)	ns
	0.7 (0.52–0.87)	1.9 (1.7–2.6)	5 (2.8–8.3)	0.0002
	0.85 (0.57–1.12)	0.9 (0.52–1.4)	5 (3.5–6.5)	0.004
Regulatory T cells:				
− Treg − Treg/Th17 − Treg/Th1	0.31 (0.23–0.38)	0.35 (0.32–0.44)	23.9 (22.4–26.5)	0.01
	0.005 (0.003–0.008)	0.12 (0.06–0.18)	1.9 (1.13–2.9)	<0.0001
	18.5 (15.6–41.9)	0.02 (0.01–0.03)	2.8 (2.8–3.4)	<0.0001

## Data Availability

The data presented in this study are available on request from the corresponding author.

## Supplementary Materials

The following are available online at https://www.mdpi.com/article/10.3390/cells10040780/s1, Figure S1: (a) Unit variance scaling is applied to rows; SVD with imputation is used to calculate principal components. X and Y axis show PC 1 and PC 2 that explain 22.3% and 17.1% of the total variance, respectively. Prediction ellipses are with a probability of 0.95. (b) Unit variance scaling is applied to rows; SVD with imputation is used to calculate principal components. X and Y axis show principal component 1 and principal component 3 that explain 22.3% and 10.7% of the total variance, respectively. Prediction ellipses are with a probability of 0.95. (c) Heatmap analysis. Rows are centered; unit variance scaling is applied to rows, Figure S2: (a) Unit variance scaling is applied to rows; SVD with imputation is used to calculate principal components. X and Y axis show principal component 1 and principal component 2 that explain 29.1% and 15.5% of the total variance, respectively. Prediction ellipses are with a probability of 0.95. (b) Unit variance scaling is applied to rows; SVD with imputation is used to calculate principal components. X and Y axis show principal component 1 and principal component 3 that explain 29.1% and 14.8% of the total variance, respectively. Prediction ellipses are with a probability of 0.9. (c) Rows are centered; unit variance scaling is applied to rows. Both rows and columns are clustered using Euclidean distance and average linkage.

## Abbreviations

Chronic allograft dysfunction	CLAD
Lung transplant	LTx
Acute cellular rejection	AR
Chronic lung allograft dysfunction	CLAD
Bronchiolitis obliterans syndrome	BOS
Restrictive allograft syndrome	RAS
T helper	Th
Bronchoalveolar lavage	BAL
Peripheral blood mononuclear cells	PBMCs
Forced vital capacity	FVC
Forced expiratory volume in the first second	FEV1
Diffusing capacity of the lung for carbon monoxide	DLCO
