# Supplementary material for: Regulatory and Effector Cell Disequilibrium in Patients with Acute Cellular Rejection and Chronic Lung Allograft Dysfunction after Lung Transplantation: Comparison of Peripheral and Alveolar Distribution
Source: Cells. 2021 Apr 1;10(4):780. doi: 10.3390/cells10040780 (PMC8065700; doi:10.3390/cells10040780)
Supplement: Supplementary file 1 [file cells-10-00780-s001.pdf]

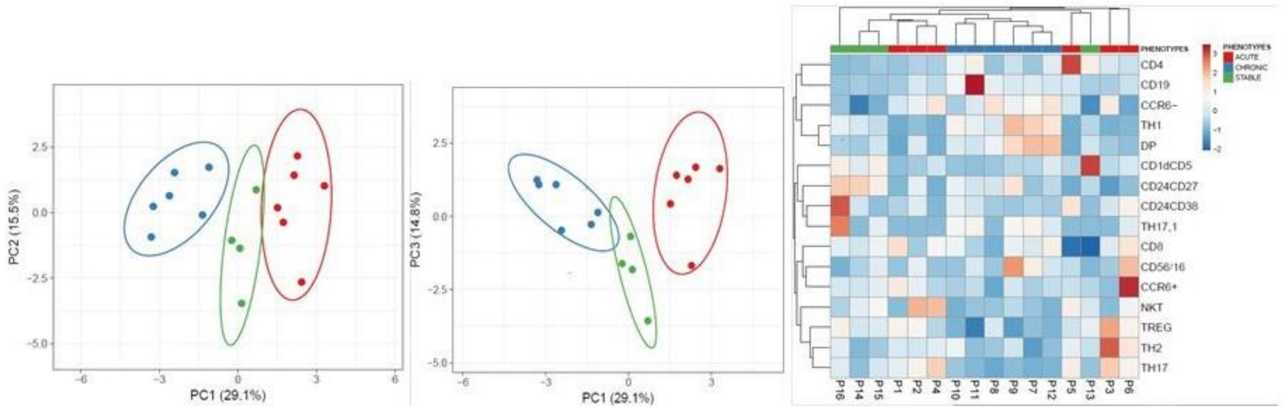

A) B) C)

Supplementary Figure 1. (a) Unit variance scaling is applied to rows; SVD with imputation is used to calculate principal components. X and Y axis show PC 1 and PC 2 that explain 22.3% and 17.1% of the total variance, respectively. Prediction ellipses are such that with probability 0.95. (b) Unit variance scaling is applied to rows; SVD with imputation is used to calculate principal components. X and Y axis show principal component 1 and principal component 3 that explain 22.3% and 10.7% of the total variance, respectively. Prediction ellipses are such that with probability 0.95 (c) Heatmap analysis. Rows are centered; unit variance scaling is applied to rows.

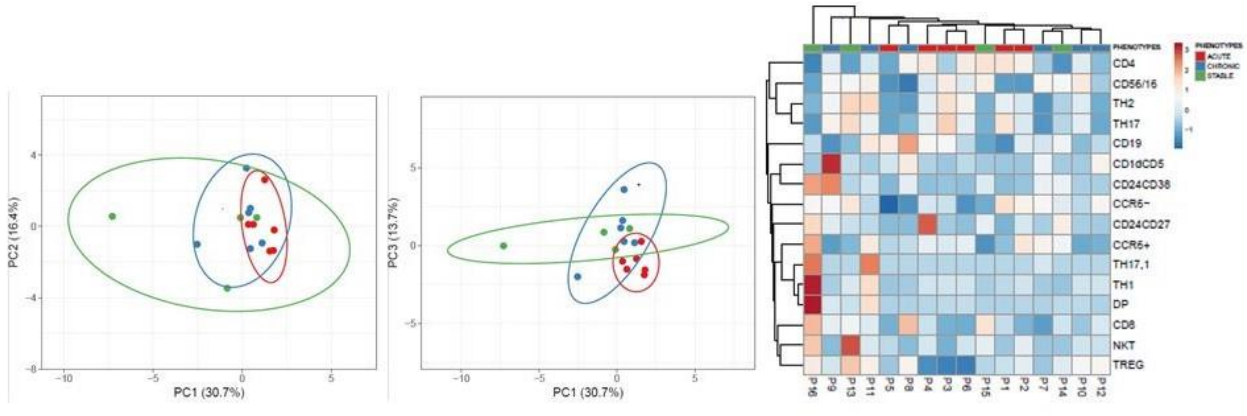

a) b) c)

Supplementary Figure 2. (a) Unit variance scaling is applied to rows; SVD with imputation is used to calculate principal components. X and Y axis show principal component 1 and principal component 2 that explain 29.1% and 15.5% of the total variance, respectively. Prediction ellipses are such that with probability 0.95 (b) Unit variance scaling is applied to rows; SVD with imputation is used to calculate principal components. X and Y axis show principal component 1 and principal component 3 that explain 29.1% and 14.8% of the total variance, respectively. Prediction ellipses are such that with probability 0.9. (c) Rows are centered; unit variance scaling is applied to rows. Both rows and columns are clustered using Euclidean distance and average linkage.
